# YDD-SLAM: Indoor Dynamic Visual SLAM Fusing YOLOv5 with Depth Information

**DOI:** 10.3390/s23239592

**Published:** 2023-12-03

**Authors:** Peichao Cong, Junjie Liu, Jiaxing Li, Yixuan Xiao, Xilai Chen, Xinjie Feng, Xin Zhang

**Affiliations:** School of Mechanical and Automotive Engineering, Guangxi University of Science and Technology, Liuzhou 545006, China

**Keywords:** dynamic VSLAM, ORB-SLAM3, YOLOv5, object classification, depth information fusion

## Abstract

Simultaneous location and mapping (SLAM) technology is key in robot autonomous navigation. Most visual SLAM (VSLAM) algorithms for dynamic environments cannot achieve sufficient positioning accuracy and real-time performance simultaneously. When the dynamic object proportion is too high, the VSLAM algorithm will collapse. To solve the above problems, this paper proposes an indoor dynamic VSLAM algorithm called YDD-SLAM based on ORB-SLAM3, which introduces the YOLOv5 object detection algorithm and integrates deep information. Firstly, the objects detected by YOLOv5 are divided into eight subcategories according to their motion characteristics and depth values. Secondly, the depth ranges of the dynamic object and potentially dynamic object in the moving state in the scene are calculated. Simultaneously, the depth value of the feature point in the detection box is compared with that of the feature point in the detection box to determine whether the point is a dynamic feature point; if it is, the dynamic feature point is eliminated. Further, multiple feature point optimization strategies were developed for VSLAM in dynamic environments. A public data set and an actual dynamic scenario were used for testing. The accuracy of the proposed algorithm was significantly improved compared to that of ORB-SLAM3. This work provides a theoretical foundation for the practical application of a dynamic VSLAM algorithm.

## 1. Introduction

In recent years, with the rapid development of mobile robot navigation technology [1], simultaneous localization and mapping (SLAM) technology, as a typical representation, has received extensive attention, particularly SLAM technology based on vision sensors. Visual SLAM (VSLAM) technology enables a robot to obtain its own location information accurately with the help of a low hardware cost and to build a map around itself to achieve autonomous navigation and obstacle avoidance.

Currently, image features are widely used in various computer vision applications, such as image feature points, corners, local structural features, etc. [2,3,4], and the visual SLAM technique is one of the typical applications that make extensive use of various types of image features. Visual SLAM is the process of the bit pose estimation and map construction in an unknown environment with its own visual sensor data. With the increasing popularity of service robots, visual SLAM technology has a wide range of applications in the field of indoor mobile robots, such as indoor service robots [5] and medical service robots [6]. At present, most of the relevant studies related to VSLAM are oriented towards static scenes. The related algorithms include the monocular visual–inertial system and GNSS–visual–inertial system algorithms proposed by Shen et al. [7,8] and the ORB-SLAM [9,10,11] series of algorithms based on ORB [12] feature points proposed by the University of Zaragoza in Spain. These algorithms can be used effectively in static scenarios, but their accuracy and robustness are low in dynamic scenarios; thus, they cannot satisfy the requirements of diverse scenarios. The root cause of the above problems is that the feature points extracted by VSLAM algorithms contain non-quantitative dynamic feature points due to the widespread existence of moving objects in dynamic scenes. Using these feature points directly to calculate the pose of a robot will lead to a large deviation in the solution. With the continuous expansion of mobile robot application scenarios, VSLAM algorithms suitable for dynamic scenarios are urgently needed. Therefore, the dynamic scenario-oriented VSLAM has become a popular topic in mobile robot navigation.

In order to solve the above problems, many researchers have taken dynamic factors into account in VSLAM algorithms and proposed dynamic VSLAM algorithms. Algorithms of this type often use geometric and deep learning methods to eliminate dynamic feature points, and deep learning methods also include image segmentation and object detection. (1) The geometric method: Lu et al. [13] extracted static feature points from highly dynamic environments based on a distributed and local random sample consensus (RANSAC) algorithm, but this algorithm fails in more static scenarios. Sun et al. [14] proposed a VSLAM algorithm for the online removal of dynamic foreground objects based on RGB-D information, which is limited and extremely time-consuming to employ in high-speed scenes due to the use of dense optical flow for tracking, and its segmentation accuracy of dynamic objects is subsequently reduced when the optical flow method is degraded. (2) Image segmentation: To improve the segmentation accuracy of VSLAM algorithms for dynamic objects further, Fu et al. [15] proposed a semantic segmentation method based on the attention mechanism (the convolutional block attention module), which could eliminate dynamic feature points, but the real-time performance was poor. To improve the real-time performance of the segmentation, Liu et al. [16] utilized the PWC-Net [17] optical flow model to predict the key frame Mask R-CNN [18] output results and used the speed of the optical flow estimation as a constraint to reduce the influence of untrained dynamic objects. However, this method only utilizes the key frame information, which leads to limited accuracy in judging the dynamic feature points for each frame. (3) Object detection: Similarly, to improve the real-time performance of the dynamic VSLAM algorithm, Yan [19] and Gokcen et al. [20] relied on the short-time YOLO object detection algorithm to eliminate all feature points in the dynamic detection box, but most of the static feature points in the dynamic detection box were also eliminated. To preserve the static feature points in the object detection box as much as possible, Gong et al. [21] retained all feature points in the static detection box when the dynamic and static detection box intersected. However, due to the large area of the object detection box, this method still retained some dynamic feature points incorrectly. As can be seen from Table 1, although the geometric method can eliminate the dynamic feature points in the environment, it is more dependent on the threshold than the deep learning method. Segmentation can accurately delineate dynamic object contours, but the real-time performance is poor. Although object detection has better real-time performance than segmentation, the detection box contains a large number of static feature points that can be used for the pose estimation, and if all of them are eliminated, the localization accuracy of the VSLAM algorithm will be reduced. When the proportion of dynamic objects involved in the application scenario is too high, the dynamic feature points will be rejected in large quantities, resulting in an insufficient number of effective static feature points to meet the system requirements, which is very likely to crash the dynamic VSLAM algorithm. To summarize, the effectiveness of using the geometric method or deep learning method to eliminate dynamic feature points alone is limited, and these approaches cannot achieve sufficient positioning accuracy and real-time performance simultaneously. Further, the advantages and disadvantages of dynamic feature point elimination directly affect the positioning accuracy and robustness of dynamic VSLAM algorithms. Therefore, dynamic VSLAM algorithms still have considerable room for improvement in terms of localization accuracy, real-time performance, and robustness.

To solve these problems, this paper proposes a novel indoor dynamic VSLAM algorithm (YDD-SLAM) based on the ORB-SLAM3 algorithm. The algorithm is applicable to a variety of typical indoor scenes, such as office and restaurant scenes, etc. In this paper, we take the indoor office scene as the main object of introduction. This algorithm introduces the YOLOv5 [22] object detection algorithm, which takes a short time, and combines the depth information to achieve the fine elimination of dynamic feature points in the detection box. Compared with the current excellent dynamic VSLAM algorithm, YDD-SLAM has excellent positioning accuracy and real-time performance. In addition, aiming at the stability problem in dynamic scenarios, it incorporates a series of feature point optimization strategies, which significantly improves the positioning accuracy and robustness of the proposed algorithm. The main contributions of our approach are as follows:

(1) To make full use of the prior information of objects, we divided common indoor objects into eight subcategories according to their motion characteristics and depth values, so as to facilitate the adoption of dynamic feature point elimination and judgment methods suitable for different objects.
(2) To solve the complex problem of the range change of the depth value of non-rigid dynamic objects, we matched the information of ‘person’, ‘head’, and ‘hand’ and the corresponding depth value in the object detection class and combined them into the depth range of ‘human body’ for the final selection of dynamic feature points, which can adapt to changes with the movement of ‘human body’.
(3) Aiming at the problem of achieving sufficient positioning accuracy and real-time performance of the dynamic VSLAM algorithm, we proposed an improved indoor dynamic VSLAM algorithm based on ORB-SLAM3 -YDD-SLAM to avoid excessive feature point elimination due to the overly large object detection box area of the object detection algorithm. This approach introduces short-time-consumption YOLOv5 and deep information fusion to achieve the fine elimination of dynamic feature points in the object detection box and to retain static feature points in the detection box as much as possible. The removal effect of dynamic feature points is close to that of image segmentation, but it can save a considerable amount of time.
(4) To solve the robustness problem of the dynamic VSLAM algorithm, we propose a series of feature point optimization strategies based on depth information and feature point extraction methods to improve its positioning accuracy and robustness in complex scenes.

## 2. Related Works

In dynamic VSLAM, the ability to remove dynamic feature points using a geometric method or deep learning method alone is limited. At present, the mainstream method is to combine a geometric method with deep learning theory and to use geometric information to process the deep learning results further. We describe this work in Section 2.1. As application scenarios continue to increase, researchers will classify feature points according to their own algorithm characteristics and application scenarios to make the dynamic VSLAM algorithm more adaptable to these situations. We will introduce relevant work in Section 2.2.

### 2.1. Method Based on Deep Learning and Geometric Information Fusion

To improve the real-time performance of dynamic VSLAM, some researchers have used lightweight object detection algorithms to recognize dynamic objects. Wang [23], Cheng [24], and Zhao et al. [25] combined a short-time object detection algorithm with epipolar geometry to judge dynamic feature points in the environment by comparing the distance between the feature points and polar lines in the detection box. However, the epipolar geometry has a limited ability to identify small displacement objects. Su et al. [26] combined the object detection algorithm with the optical flow method to judge dynamic feature points and determined the elimination range of the dynamic feature points by calculating the optical flow value of the feature points in the detection box. However, the optical flow method had high illumination brightness requirements. To make full use of sensor information, some researchers have combined deep learning with camera depth information. The DynaSLAM algorithm, proposed by Bescos et al. [27], and the WF-SLAM algorithm, proposed by Zhong et al. [28], are both based on the combination of a Mask R-CNN segmentation network and multi-view geometry and adopt the multi-view geometry method based on the depth threshold to make further dynamic judgments regarding the segmentation feature point results. However, both algorithms are extremely time-consuming. SOLO-SLAM, proposed by Sun et al. [29], also uses image segmentation with multi-view geometry for the rejection of dynamic feature points, and the algorithm utilizes the depth error of the map points for the further rejection of these points. Yang et al. [30] adopted a motion consistency algorithm combining spatial structure relation, depth information, and combined semantic segmentation to achieve the pose estimation in a dynamic environment. However, this algorithm is also time-consuming. The Det-SLAM algorithm, proposed by Eslamian et al. [31], combines the semantic segmentation network and depth information to remove dynamic feature points, but this method considers the depth information of all regions in the image bounding box, which further increases the computational cost. To improve the accuracy of VSLAM algorithms in dynamic scenarios, Tian et al. [32] proposed a method of identifying the range of dynamic objects based on the object detection algorithm. In a specific range, the depth information of the current frame and the multi-view geometry information of the key frame are combined to screen dynamic feature points. However, as the feature points may not have the corresponding matching relationship in the two frames, this affects the judgment of the dynamic feature points. The dynamic VINS, proposed by Liu et al. [33], combines the object detection algorithm with the depth information to achieve the effect of approaching image segmentation. The algorithm uses the depth of the central position of the person detection box and the depths of four corner points to judge the range of dynamic feature points, but the depth information is not fully utilized by this algorithm. The center of the person detection box is sometimes not on the human body, which affects the robustness of the algorithm. Wang et al. [34] proposed a dynamic environment-oriented FCH-SLAM algorithm, which also combined semantic information and depth information to eliminate dynamic feature points, but the algorithm did not consider potentially dynamic objects other than ‘human body’. In summary, dynamic VSLAM algorithms using segmentation methods are generally time-consuming. Although the dynamic VSLAM algorithm using the lightweight object detection algorithm can satisfy the real-time requirements, the geometric method with which it is integrated either has high requirements on the environment or has defects in the judgment of dynamic feature points. Some researchers adopt the method of the fusion of deep information and deep learning to eliminate dynamic feature points, but their use of deep information is simple and insufficient. To solve the above problems, an indoor dynamic VSLAM algorithm combining YOLOv5 and depth information was developed in this study, which is called YDD-SLAM. Compared with the above work, our dynamic VSLAM algorithm can make full use of object depth information and has good accuracy and real-time performance.

### 2.2. Feature Point Classification

In dynamic VSLAM algorithms, to utilize the prior information of objects fully, researchers customize the classification of environmental objects, and the most common approach is to divide objects in dynamic environments into dynamic, potentially dynamic, and static objects. Bahraini et al. [35] used the multilevel RANSAC method to classify objects in a dynamic environment into static and moving categories and to track moving objects. However, the computational complexity of this method was relatively high. Cui et al. [36] used SegNet [37] to divide scene objects into static, dynamic, and potentially dynamic objects and eliminated dynamic feature points by tightly coupling semantic and geometric information. However, this method was time-consuming. To ensure the smooth transition of the VSLAM algorithm from static to dynamic scenes, Barsan et al. [38] used instance segmentation and sparse scene flow to divide dynamic scenes into background, moving objects, and potentially moving objects. Due to the use of instance segmentation, the real-time performance of this method is low. Ran et al. [39] and Hu et al. [40] divided dynamic object points into dynamic and movable object points, used static object points for mapping, and employed static and movable object points for front-end tracking to obtain more accurate tracking results. To solve the special problems posed by their own algorithms and to utilize the environment information more fully, when classifying the objects in the dynamic environment into dynamic, potentially dynamic, and static objects can no longer meet the requirements, some researchers further classify the objects according to the characteristics of the problems to be solved by their own algorithms. To solve the initialization problem of the dynamic VSLAM algorithm in plane and non-plane cases, Wen et al. [41] used the double projection error method based on attribute constraints to divide feature points into dynamic and static types and, further, to divide them into plane and non-plane types. However, this method is prone to generate image motion blur and weak texture problems. Yang et al. [42] divided the objects in a scene into two categories: static and dynamic groups. Then, they subdivided the static group into ‘Permanent Structures’ and ‘Furniture’, subdivided the dynamic group into ‘Fixed Objects’, ‘Electrical Appliances’, and ‘Props’, and assigned different weights according to the motion properties of the objects. Dynamic object information is used to estimate poses by fusing semantic information and motion attributes. The proposed algorithm has good positioning accuracy and robustness in high dynamic scenes. In summary, the classification of objects in dynamic environments into dynamic, potentially dynamic, and static categories can satisfy the requirements of most dynamic VSLAM algorithms, but this classification method cannot meet the requirements of dynamic VSLAM algorithms to solve some special problems. To make the classified objects meet the requirements of use, some researchers conduct a secondary classification based on the above classification according to the characteristics of the problems to be solved by their algorithms. Inspired by the above related work, the YDD-SLAM algorithm presented in this paper first divides dynamic environment objects into three categories: dynamic, potentially dynamic, and static. In this study, multiple depth information of different objects was used to judge and remove dynamic feature points, and the precision of the object depth range directly affects the removal effect of the proposed algorithm. If the objects are only divided into the above three categories, the depth range of the calculated dynamic feature points will be too large, due to the insufficient use of the depth information of the objects, which will affect the ability to eliminate the dynamic feature points in the developed approach. To improve the accuracy of the depth range used to judge dynamic feature points, common indoor objects are divided into eight subcategories in this paper, based on the above three categories according to the characteristics of the algorithm and the motion characteristics and depth values of indoor objects.

## 3. Materials and Methods

### 3.1. Overview of the YDD-SLAM System

**Figure 1:**
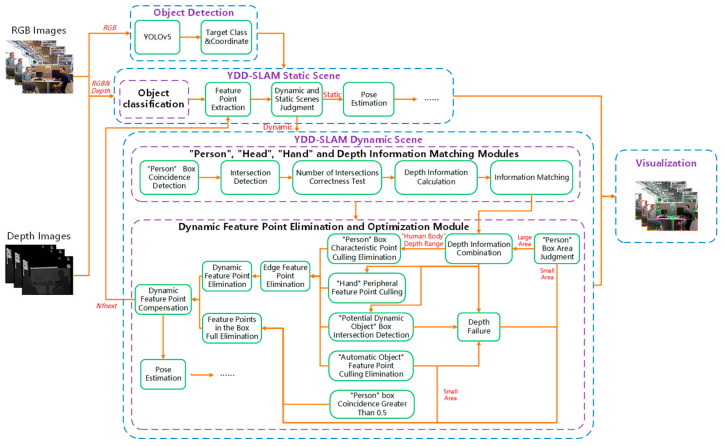
YDD-SLAM system block diagram.

The ORB-SLAM3 algorithm involves tracking, local mapping, back-end optimization, and visualization. It supports various camera and inertial measurement unit (IMU) combination modes. This paper proposes a VSLAM algorithm for indoor dynamic scenes, which is called YDD-SLAM. The algorithm is improved based on ORB-SLAM3; the YOLOv5 object detection algorithm is introduced, and the depth information is integrated to identify and eliminate dynamic feature points. The YDD-SLAM algorithm mainly provides improved tracking and visualization compared to those of ORB-SLAM3. It is divided into four parts: object detection, YDD-SLAM static scene, YDD-SLAM dynamic scene, and visualization. The YDD-SLAM dynamic scene includes the ‘person’, ‘head’, ‘hand’, and depth information matching modules, as well as the dynamic feature point elimination and optimization module, as shown in Figure 1.

(1) Object detection: To balance the detection speed and accuracy of the YOLOv5 object detection algorithm, the YOLOv5s model was used for object detection in this study. The RGB image is passed to the YOLOv5 object detection algorithm. The detection box is coordinated, and class name results are passed to the YDD-SLAM algorithm.
(2) YDD-SLAM dynamic and static scenes: According to the class name information passed by YOLOv5, the YDD-SLAM algorithm divides indoor objects into three categories: dynamic, potentially dynamic, and static. On this basis, objects are subdivided into eight subcategories. Then, the total number of feature points to be extracted is calculated by the dynamic feature point compensation strategy of the previous frame, and the feature points are extracted according to the number requirements of the current RGB image frame. Next, according to the object classification information, the action and static scene of the current image frame are judged. If no ‘dynamic object’ information is detected in the image for five or more consecutive frames, the current image frame is considered to be a static scene, and the pose estimation in the static scene is consistent with the ORB-SLAM3 algorithm. Otherwise, the current image frame is considered to be a dynamic scene, which needs to match the information of ‘person’, ‘head’, ‘hand’, and depth. The matching results are transmitted to the dynamic feature point elimination and optimization module, and the feature points are dynamically judged and eliminated. The static feature points are used for the pose estimation.
(3) Information matching module: Firstly, the coincidence degree of the ‘person’ detection box in the current image frame is calculated. When the coincidence degree of the ‘person’ box is greater than 50%, the dynamic feature point elimination and optimization module adopts the strategy of all feature points in the frame. The ‘person’ box with a coincidence degree less than 50% matches the ‘person’, ‘head’, ‘hand’, and depth information. Secondly, the intersection detection of the ‘person’ box information and the ‘head’ and ‘hand’ boxes of the current frame is conducted, and the correctness judgment is made according to the number of ‘person’, ‘head’, and ‘hand’ boxes. If the number is correct, the corresponding calculated depth information is directly matched and attached. When the number of intersections is abnormal, matching must be performed according to the geometry and depth information.
(4) Dynamic feature point elimination and optimization module: Firstly, the area of the ‘person’ box is judged; the ‘person’, ‘head’, ‘hand’, and depth matching information are carried out on the large area ‘person’ box; and the depth information of the ‘human body’ related categories is combined to calculate the final depth range of the ‘human body’. Secondly, the dynamic character of the ‘potentially dynamic object’ is judged. Then, the feature points in the box of ‘dynamic object’ and ‘potentially dynamic object’ are combined with the depth range information to eliminate the dynamic feature points. When the depth value of the feature points in the box is within this depth range, the feature points are determined to be dynamic feature points and eliminated. Meanwhile, the feature points around the ‘hand’ and the edge feature points are eliminated. The strategy of eliminating all feature points in the detection box was adopted for the three cases of small area ‘human’ box, the object with failed depth, and the ‘human’ box with a coincidence degree greater than 50%. Finally, the static feature points are used for the pose estimation, and the total number of feature points to be extracted in the next frame is calculated by the dynamic feature point compensation strategy and passed to the feature point extraction part.
(5) Visualization: Different colored boxes are used to visualize the object detection results of various objects. Simultaneously, the central position of the ‘person’ box is marked, the dynamic feature points are removed and not displayed, and the retained static feature points are displayed.

### 3.2. Indoor Object Classification

Aiming at the real-time problem of the dynamic VSLAM algorithm, to avoid too much feature point elimination due to the excessively large object detection box area of the object detection algorithm, the YOLOv5 object detection algorithm and image depth information were fused with the ORB-SLAM3 algorithm in this study. To combine the depth information to eliminate the dynamic feature points in the object detection box more effectively, and to make full use of the depth information and object information, common indoor objects were subdivided into eight subclasses according to the motion characteristics and depth values.

The algorithms in this paper are oriented to indoor dynamic scenes, in order to meet the common object classification needs of many typical indoor scenes. In this study, indoor objects were divided into three categories: (A) ‘dynamic objects’, (B) ‘potentially dynamic objects’, and (C) ‘static objects’. The object detection results were combined with the depth information to retain the static feature points in the dynamic detection box. To make use of the prior depth information of objects more effectively, the three categories were further subdivided into eight subcategories according to the motion characteristics and depth values of indoor objects. Specifically, the ‘dynamic objects’ category was subdivided into (D) ‘human body’ and (E) ‘automatic object’. The ‘human body’ category was subdivided into (F) ‘person’, (G) ‘head’, and (H) ‘hand’. Meanwhile, the ‘potentially dynamic objects’ category was subdivided into three subcategories according to the object depth: (I) ‘small depth object’, (J) ‘medium depth object’, and (K) ‘large depth object’.

In the indoor environment, the human body is usually the largest and most complex dynamic factor, so ‘human body’ was set as the ‘dynamic object’ category. The human body is a non-rigid object, and the variable posture of the human body leads to the complex and constantly changing depth range of the object. Because the head can be seen in most cases as the middle of the depth of the human body, a potentially dynamic object is moved in most cases by the action of the hand. To facilitate the acquisition of the depth range information of the human body and the judgment of the movement of the potential dynamic objects, ‘human body’ was subdivided into three subcategories: ‘person’, ‘head’, and ‘hand’. By calculating the depth ranges of these three types of objects, the depth range of the human body was finally combined to judge and eliminate the dynamic feature points in the person. ‘Automatic object’ refers to an object that can move by itself without the action of the human body, such as a fan that can be turned.

Some indoor objects usually undergo pose changes under the action of the human body. In this study, we defined such objects as ‘potentially dynamic objects’, which are at rest most of the time. We compared the depth range of the object with the depth value of the feature point in the dynamic detection box to determine whether the feature point is a dynamic feature point. To make full use of the prior depth information of the object, ‘potentially dynamic objects’ were subdivided into ‘small depth objects’, ‘medium depth objects’, and ‘large depth object’ according to the object depth, as mentioned previously. In this study, the depth range of ‘small depth objects’ was set as 0–20 cm, the depth range of ‘medium depth objects’ was set as 20–45 cm, and the depth range of ‘large depth objects’ was set as 45–75 cm (see Equation (1) for details). The depth range and classification number of ‘potentially dynamic objects’ can be reasonably set according to their own usage scenarios.

(1) $$\left\{\begin{array}{c} ‘\mathrm{small}\;\mathrm{depth}\;\mathrm{object}’,\;0<x\leq 20\\ ‘\mathrm{medium}\;\mathrm{depth}\;\mathrm{object}’,\;20<x\leq 45\\ ‘\mathrm{large}\;\mathrm{depth}\;\mathrm{object}’,\;45<x\leq 70 \end{array}\right.$$

In this study, the objects not in the above two categories of ‘dynamic objects’ and ‘potentially dynamic objects’ were set as ‘static objects’. Generally, a static object will not be moved by the human body, so the extracted feature points of this kind of object are static feature points, which can be used for the pose estimation in the VSLAM algorithm.

### 3.3. Information Matching

#### 3.3.1. Appropriate Minimum Depth of Object

In this study, eight types of objects were detected, and the dynamic feature points in the dynamic detection box were identified with depth information. Different kinds of objects have different shapes and different depth value extraction methods.

**Figure 2:**
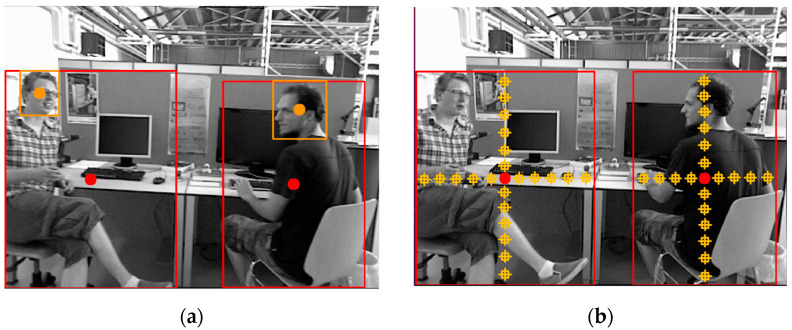
Object center position and depth information extraction. The red box is the ‘person’ detection box, and the red circle is its center position. (**a**) Center of object; (**b**) Approximate minimum depth acquisition schematic. The yellow box is the ‘head’ detection box, and the yellow circle is its center position. The shape of the combination of the small yellow box and the cross represents the sketch of the pixel points selected to calculate the depth in this paper.

As the center of the detection box of the head is basically located on the object, as shown in Figure 2a, the approximate minimum depth value of the head only needs to obtain the depth value of the center position. The morphological diversity of ‘person’, ‘hand’, ‘automatic object’, and ‘potentially dynamic object’ leads to the fact that the central position of the detection box is not necessarily on the object. Therefore, a certain number of pixels from the upper, lower, left, and right positions of the center of the object detection box were employed in this study to extract the depth value, as shown in Figure 2b. Firstly, with the center of the detection box as the boundary, the depth values extracted from the left and right sides of the center point were summed and compared, the depth values on the smaller side were sorted, and the first three minimum depth values were taken and their average values were calculated. The same process was performed for the depth values of the top and bottom sides, and the mean was calculated. Finally, the above two means were compared, and the depth with the smaller mean was regarded as the approximate minimum depth of the object. All the object depth values mentioned below refer to the approximate minimum depth of the object.

#### 3.3.2. ‘Person’, ‘Head’, ‘Hand’, and Depth Information Match

In this study, ‘human body’ was subdivided into three subcategories: ‘person’, ‘head’, and ‘hand’, in order to obtain the final depth range of the ‘human body’ and the motility judgment of ‘potentially dynamic objects’. Because the output results of the object detection are disordered, the object detection results of ‘person’, ‘head’, and ‘hand’ must be matched before they can be used.

**Figure 3:**
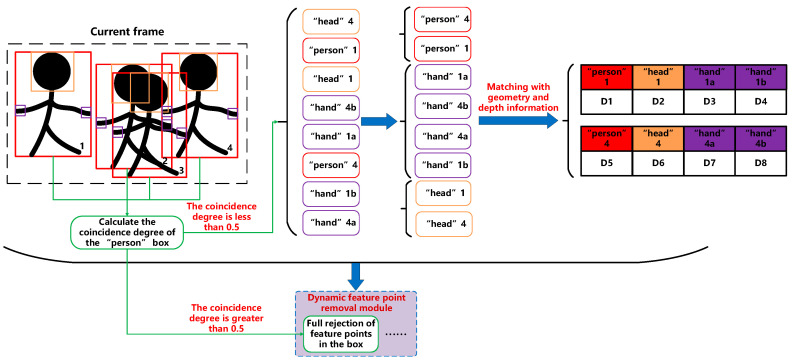
‘Person’, ‘head’, ‘hand’, and depth information match.

**Figure 4:**
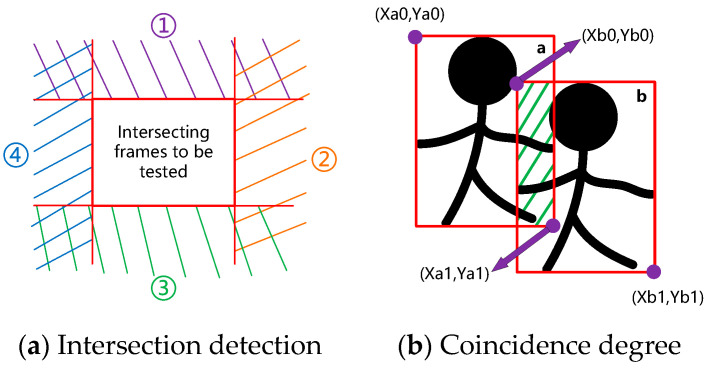
Intersection detection and coincidence degree. ①–④ corresponds to the four inequality cases in Equation (2).

As shown in Figure 3, object detection is firstly performed on the current frame image, and the identified information about the ‘person’, ‘head’, and ‘hand’ is classified and stored. Then, the intersection detection and coincidence degree calculations of all ‘person’ boxes are conducted, as depicted in Figure 4. The four cases in Equation (2) correspond to the four ranges in Figure 4a. When the two detection boxes do not satisfy any relation in the equation, they are judged to intersect; conversely, when any relation is satisfied, the two detection boxes are judged to be disjointed. The coincidence degree is calculated as shown in Figure 4b and Equation (3). Sa and Sb are the areas of the two detection boxes, respectively, Sd is the area of the coincidence part of the two detection boxes, and Ora and Orb are the coincidence degrees of the two detection boxes themselves.

Intersection detection:

(2) $$\left\{\begin{array}{c} Xb1<Xa0\\ Yb1<Ya0\\ Xb0>Xa1\\ Yb0>Ya1 \end{array}\right.$$

Calculation of the coincidence degree:

(3) $$Sa=(Xa1-Xa0)\cdot (Ya1-Ya0)\;Sb=(Xb1-Xb0)\cdot (Yb1-Yb0)\;X0=max(Xa0,Xb0)\;X0=max(Xa0,Xb0)\;Y0=max(Ya0,Yb0)\;X1=max(Xa1,Xb1)\;Y1=max(Ya0,Yb0)\;Sd=(X1-X0)\cdot (Y1-Y0)\;Ora=\frac{Sd}{Sa\;}=\frac{\left(X1-X0)\cdot (Y1-Y0\right)}{\left(Xa1-Xa0)\cdot (Ya1-Ya0\right)}\;Orb=\frac{Sd}{Sb\;}=\frac{\left(X1-X0)\cdot (Y1-Y0\right)}{\left(Xb1-Xb0)\cdot (Yb1-Yb0\right)}$$

Firstly, the intersection detection of the ‘person’ box is conducted. If the ‘person’ box does not intersect with other ‘person’ boxes in the current frame, the coincidence degree is 0. If the ‘person’ box intersects with other ‘person’ boxes, the coincidence degree is calculated. In the ‘person’ box with a coincidence degree greater than 50%, matching the information is difficult because of information redundancy. Therefore, we adopted a total elimination strategy for the feature points in the above ‘person’ box. If the coincidence degree is not 0 and less than 50%, the ‘hand’ box inside the ‘person’ box is not matched, because in the ‘person’ box with coincidence, the number of ‘hand’ is large and the position is complex, so only the information of ‘person’ and ‘head’ is matched in this case. Next, the intersection between ‘person’ and ‘hand’ boxes is checked. If the number of intersections between a ‘person’ and ‘hand’ box is 0 (no head is detected) or 1, it is normal and can be matched directly. When the number of intersections between a ‘person’ box and a ‘head’ box is greater than 1, a further judgment is needed to match. Considering this situation, the proposed approach uses the combination of geometry and depth information to make the matching judgment. Firstly, when the aspect ratio of the ‘person’ box is less than 1, that is, the box is vertically long, whether the ‘head’ box is located in the upper part of the ‘person’ box is judged. Secondly, the depth values of ‘person’ and ‘head’ are obtained for comparison, and the depth difference between the two must meet a certain threshold, so as to avoid the mismatching problem caused by the misdetection of background objects and the coincidence of the background ‘person’. According to the above judgment, if the number of ‘head’ boxes satisfying the conditions is still greater than 1, the difference of the pixel distance on the border of all ‘head’ and ‘person’ boxes satisfying the conditions should be calculated, and the ‘head’ with the smallest difference should be matched with the ‘person’ box.

If the coincidence ratio of the ‘person’ box is 0, the ‘person’ box does not intersect other ‘person’ boxes. In this case, the information of ‘person’, ‘head’, and ‘hand’ must be matched. The matching mode of ‘person’ and ‘head’ is the same as that of the preceding matching mode when the coincidence degree is not 0 but less than 50%. The information matching of ‘person’ and ‘hand’ is also similar to that of ‘head’. The intersection detection of ‘person’ and ‘hand’ boxes is also performed firstly. If the number of intersections between a ‘person’ box and a ‘hand’ box is 0 (no ‘hand’ is detected), 1 (only 1 ‘hand’ is detected), or 2, it is normal and can be directly matched. When the number of intersections between a ‘person’ box and a ‘hand’ box is greater than 2, a further judgment is required for matching. If the ‘person’ and ‘head’ boxes of the previous step are successfully matched, the depth of the ‘person’ is represented by the depth of the ‘head’ that is successfully matched, because the ‘head’ depth value can represent the middle value of the ‘person’ depth range well. The depth of the ‘head’ and depth of the ‘hand’ are compared, and the depth difference between the two must meet a certain threshold to match successfully. If the ‘person’ and ‘head’ boxes in the previous step are not matched, the depth values of ‘person’ and ‘hand’ are compared. The depth difference between the two boxes must meet a certain threshold to be matched successfully. Because the matching process of ‘person’, ‘head’, and ‘hand’ information uses their respective depth information, the depth information is also matched and stored with the corresponding class, which is convenient for the subsequent calculation of the depth range of ‘dynamic object’ and the removal of dynamic feature points.

### 3.4. Dynamic Feature Point Elimination

In a dynamic environment, the traditional VSLAM algorithm has low precision and poor robustness; however, introducing deep learning theory can solve these problems effectively. At present, most dynamic VSLAM algorithms cannot guarantee both location accuracy and real-time performance. To solve the above problems, the proposed approach adopts YOLOv5, which takes a short time to identify classified objects but has a dynamic object detection box that contains both static and dynamic feature points. Deleting all the feature points in the detection box will reduce the pose estimation accuracy of the VSLAM algorithm, considering that a significant depth difference exists between the dynamic object and static background. Based on this feature, the proposed approach further judges the dynamic feature points in the detection box, removes them by combining the object depth information, retains the static feature points as much as possible, and uses them for the pose estimation in the VSLAM algorithm.

#### 3.4.1. ORB-SLAM3 Fusion with YOLOv5

**Figure 5:**
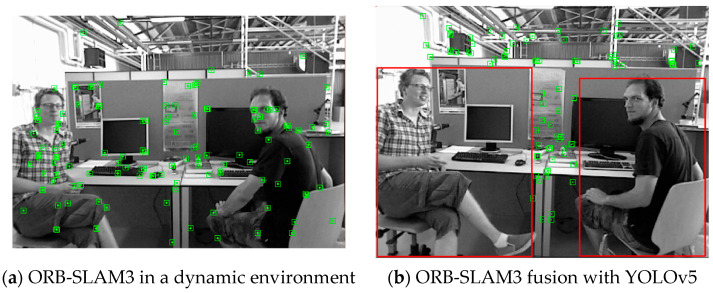
Comparison of dynamic and static VSLAM algorithms. The green squares represent the feature points, and the red squares represent the ‘person’ detection results.

Instance segmentation, semantic segmentation, and object detection are the most commonly used deep learning methods in dynamic VSLAM algorithms. Considering that the VSLAM algorithm has high real-time requirements and that segmentation takes a longer amount of time than object detection, the proposed approach adopts the fusion of YOLOv5 object detection and ORB-SLAM3 to eliminate dynamic feature points, as shown in Figure 5.

**Figure 6:**
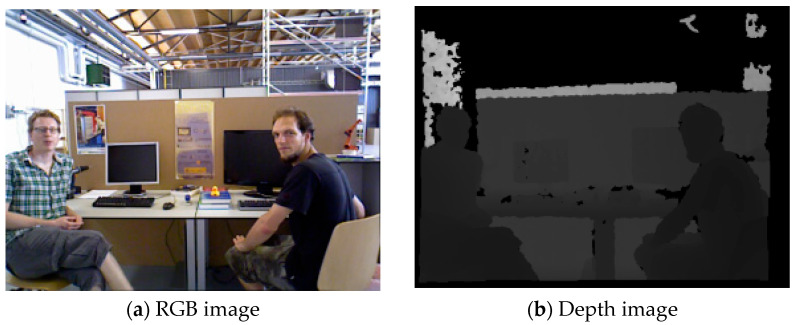
Depths of moving and static objects.

As demonstrated by Figure 5b, although YOLOv5 can detect dynamic object objects well, many feature points in the detection box are static feature points. If all feature points in the box are eliminated, the static feature points available for the pose estimation will be greatly reduced, thus affecting the accuracy and robustness of the pose estimation of the VSLAM algorithm. To solve the above problems, the proposed approach combines the YOLOv5 object detection algorithm with image depth information to refine the range of dynamic feature points in the detection box. This method utilizes the obvious depth difference between the dynamic feature points in the detection box and the static background feature points, as shown in Figure 6. The static feature points selected in the dynamic box are also used for the pose estimation in the VSLAM algorithm to meet the usage requirements of the dynamic environment.

#### 3.4.2. Feature Point Removal of ‘Dynamic Object’

Many static feature points exist in the dynamic object detection box. To retain these static feature points and remove dynamic feature points as much as possible, the depth values of the feature points were compared with the depth range of the objects in this study. When the depth value of the feature points was within the depth range of such objects, the feature points were regarded as dynamic feature points and eliminated. Table 2 lists the constants of the prior object depth value used in this study. Dhead and Dhand represent the object depths of ‘head’ and ‘hand’, respectively, and Dhalfhand represents the distance from the center point of ‘head’ to the depth of the expanded elbow. ‘Small depth object’, ‘medium depth object’, and ‘large depth object’ represent the maximum depth values of objects set as small, medium, and large depth objects, respectively.

Due to the non-rigid structure of the human body, the range of depth values of the human body is complex and constantly changing. If the depth range of the human body is set at a range that is too large, too many static feature points in the detection box will be eliminated. If the depth range is too small, some dynamic feature points will be retained. After synthesizing the above advantages and disadvantages, the depth values extracted from the subdivisions of ‘person’, ‘head’, and ‘hand’ in this study were expressed as the depth range of the human body (see Equation (4)). Then, the above three groups of depth ranges were combined into the maximum interval, which represented the final depth range of the human body, and the feature points in the dynamic detection box were eliminated and retained by using this range. Firstly, the depth of the ‘dynamic object’ described in Section 3.3.1 was obtained. Then, the ‘person’, ‘head’, ‘hand’, and depth information matched in Section 3.3.2 was determined. The depth range was calculated according to their characteristics. In Equation (4), ${Dhand1}^{\prime }$ and ${Dhand2}^{\prime }$ represent the depth values of the two hands corresponding to the ‘person’ box; Dhand1 and Dhand2 are the smaller and larger depth values of the two hands, respectively; and ${Dhead}^{\prime }$ and ${Dperple}^{\prime }$ represent the depth values of the head and person, respectively. The depth range of the human body is located between the two extended elbows most of the time, and the centre position of the head is located approximately in the middle of the human body in most cases, so Dhalfhand can be added or subtracted from the centre depth value of the head to obtain the base depth range of the human body. When the head is not detected, or the depth range of the human body exceeds the base depth range, the range of the two hand depth calculations or the range of the person depth calculations can be combined to compensate for the base depth range so that the compensated depth range can better represent the final depth range of the human body. Because the depth of each object obtained is the approximate minimum depth rather than the minimum depth, when the approximate minimum depth value is used to represent the boundary of the minimum depth value of the object, the approximate minimum depth value should be compensated. In this study, the approximate minimum depth value was subtracted from 0.2 times the maximum depth value of the object for compensation, so as to represent the minimum depth value of the object.

(4) $$Dhand1=min({Dhand1}^{\prime },{Dhand2}^{\prime })\;Dhand2=max({Dhand1}^{\prime },{Dhand2}^{\prime })\;Dheadmin={Dhead}^{\prime }+\frac{Dhead}{2}-Dhalfhand\;Dheadmax={Dhead}^{\prime }+\frac{Dhead}{2}+Dhalfhand\;Dhandmin=Dhand1+0.2\cdot Dhand\;Dheadmax=Dhand2+Dhand\;Dperplemin={Dperple}^{\prime }-0.2\cdot 2\cdot Dhalfhand\;Dperplemax={Dperple}^{\prime }+2\cdot Dhalfhand\;\left\{\begin{array}{c} \left[Dheadmin,Dheadmax\right],\;‘\mathrm{head}’\\ \left[Dhandmin,Dhandmax\right],\;\;‘\mathrm{hand}’\\ \left[Dperplemin,Dperplemax\right],\;‘\mathrm{person}’ \end{array}\right.$$

**Figure 7:**
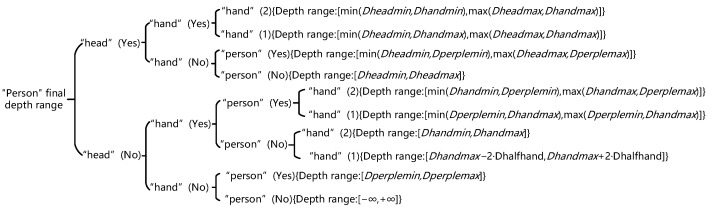
‘Human body’ final depth ranges in various cases.

In this study, the combination of the depth intervals of ‘person’, ‘head’, and ‘hand’ was classified and discussed according to various possible dynamic object situations. As the depth interval calculated by using the head can better represent the depth range of the human body, and in order to improve the calculation efficiency and accuracy, the depth interval of the head was chosen whenever possible. When there was no ‘head’ box in the ‘person’ box or the depth value of ‘head’ was invalid, the depth intervals of the ‘person’ and ‘hand’ boxes were selected. Simultaneously, in each case, at most two kinds of depth intervals of ‘person’, ‘head’, and ‘hand’ were selected to combine the final depth range of the human body, as shown in Figure 7.

To avoid increasing the category and complexity of the object, the depth range of the automatic object was calculated according to the maximum depth value of the large depth object (see Equation (5), where ${Dauto}^{\prime }$ is the depth value of the automatic object). Finally, the depth values of the feature points in the detection boxes of ‘person’ and ‘automatic object’ were compared with the depth range of the object. When the depth values of the feature points were within the depth range of such objects, the feature points were regarded as dynamic feature points and were eliminated.

(5) $$\left[{Dauto}^{\prime }-0.2\cdot Dlage,{Dauto}^{\prime }+Dlage\right]$$

#### 3.4.3. Feature Point Removal of ‘Potentially Dynamic Object’

A potentially dynamic object usually moves under the action of the hand, so when a hand in the ‘person’ box intersected with a potentially dynamic object and the depth values of the two were similar, the potentially dynamic object was regarded as a dynamic object.

**Figure 8:**
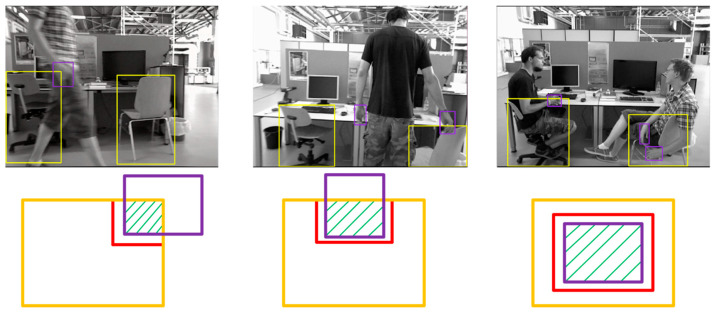
Three cases of ‘hand’ intersecting with ‘potentially dynamic objects’. The yellow square is the detection box for ‘large depth object’, and the purple square is the detection box for ‘hand’. The red color is the area of extended pixels, and the slash line indicates the area where ‘hand’ and ‘large depth object’ coincide.

When the ‘hand’ box and the ‘potentially dynamic object’ box intersected, the coincidence part of the two was extracted. If the potentially dynamic object was displaced under the action of the hand, the adjacent pixels of the coincidence box were usually pixels of the potentially dynamic object. By comparing whether the depth values of the adjacent pixels and the hand were similar, we could determine whether the object was moved by the hand. Firstly, the parts of the four sides of the coincidence box that did not coincide with the detection box of the potentially dynamic object were extracted, and the non-coincidence edge was extended outward by 3 pixels, as shown in the red box in Figure 8. When the distance between the edge of the coincidence box that needed to be extended and the detection box of the potentially dynamic object was less than 3 pixels, the edge of the coincidence box that needed to be extended only extended outward by 1 pixel. When three or more of the depth values of the extended edge were within the depth range of the hand and the depth value of the hand was within the depth range of the potentially dynamic object, the potentially dynamic object was regarded as a dynamic object.

When the object detection algorithm did not detect a hand in the ‘person’ box or only detected a hand, for instance, if the hand was blocked or missed by the potentially dynamic object, even if the potentially dynamic object had no intersection with the ‘hand’ box but did have an intersection with the ‘person’ box, the object could also have motion. In this case, the final depth range of the human body was compared with the depth of the potentially dynamic object using the approach described in Section 3.4.2. If the depth values were similar, the potentially dynamic object was considered a dynamic object.

When the ‘potentially dynamic object’ was judged to be a dynamic object, the feature points on the object were removed according to the maximum depth range set for the object as described in Section 3.2, and the background feature points were retained as much as possible. Firstly, the depth value of the potentially dynamic object was obtained according to the approach described in Section 3.3.1. Then, the depth range was calculated according to the maximum depth of the class. For details, see Equation (6), where d represents the maximum depth value set for the potentially dynamic object and $D^{\prime }$ represents the depth value extracted for the potentially dynamic object. When the depth value of the feature point in the box belonged to the depth interval of the object, the feature point was regarded as a dynamic feature point and was eliminated.

(6) $$\left\{\begin{array}{c} d=\mathrm{Dsmall},\;‘\mathrm{small}\;\mathrm{depth}\;\mathrm{object}’\\ d=\mathrm{Dmedium},\;‘\mathrm{medium}\;\mathrm{depth}\;\mathrm{object}’\\ d=\mathrm{Dlage},\;‘\mathrm{large}\;\mathrm{depth}\;\mathrm{object}’ \end{array}\right.\;\left[D^{\prime }-0.2\cdot d,D^{\prime }+d\right]$$

### 3.5. Dynamic VSLAM Feature Point Optimization Strategy

In a dynamic environment, the depth information of the object can be compared with the depth value of the feature point in the detection box, so as to judge whether the feature point is a dynamic feature point and whether it can be removed. Although the dynamic feature points can be successfully eliminated, the dynamic VSLAM algorithm still has some problems that prevent it from being optimized. For example, a pixel point on a dynamic object may become a condition for judging the edge feature point, and when the dynamic object moves, the feature point may lose the condition for being a feature point and become a redundant feature point. Alternatively, when the dynamic feature points are eliminated by the dynamic VSLAM algorithm, the static feature points used for the pose calculation will be reduced. In highly dynamic scenarios, too many dynamic feature points are eliminated, which easily leads to the pose tracking failure of the VSLAM algorithm. In dynamic scenes, some potentially dynamic objects exist that are too small to be detected, which affects the removal of dynamic feature points. In some complex dynamic scenarios, the removal conditions of the dynamic feature points fail, and the dynamic feature points are not easy to judge. As the above problems affect the positioning accuracy and robustness of the dynamic VSLAM algorithm, this paper proposes a series of dynamic VSLAM feature point optimization strategies to solve these issues.

#### 3.5.1. Edge Feature Point Elimination

**Figure 9:**
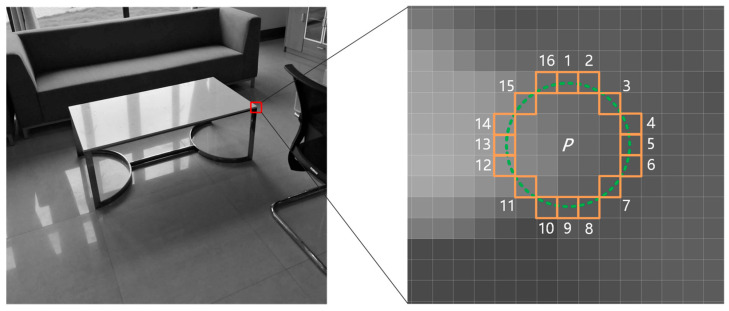
Fast corner extraction. The small orange squares represent the 16 pixels around pixel P, which are 3 pixels away from pixel P.

ORB-SLAM3 extracts ORB [12] feature points based on the fast corner detection algorithm, as shown in Figure 9. The algorithm extracts feature points by comparing the brightness of pixels with a radius of 3 pixels around the object pixel. Each layer of the pyramid is to be extracted for a certain proportional number of ORB feature points, as described in the next subsection. Considering the variable position of the moving object, when the extracted feature point is within 3 pixels from the edge of the object, the feature point may use the brightness of the pixel point on the dynamic object as part of the condition. When the object leaves the original position, the feature point may lose the condition necessary to become a feature point, resulting in the instability of the feature point.

To solve the instability problem of feature points around the above moving objects, and considering that the detection box error and unstable feature points may be outside the edge of the box, the proposed approach expands the detection box of YOLOv5 by 3 pixels to the periphery to include the unstable feature points around the moving objects. To detect unstable feature points on the edges of moving objects, the developed method uses the static feature points retained in the dynamic box to screen and uses 3 pixels as the radius to judge. To speed up the calculation, only the depth values of 4 pixels with a distance of 3 pixels from the feature points in the horizontal and vertical directions are extracted. The positions of four pixels (1, 5, 9 and 13) in Figure 9 are used to determine whether the depth of the four pixels is within the depth range of the moving object. As long as one or more pixel depth values meet the conditions, the feature point is judged to be unstable and eliminated.

#### 3.5.2. Dynamic Feature Point Compensation

**Figure 10:**
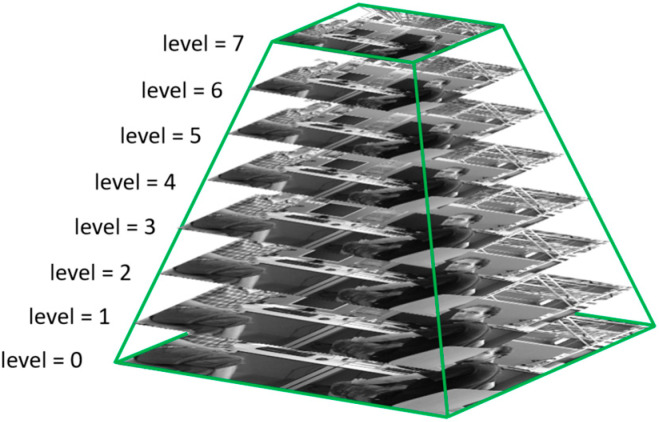
Image pyramid.

To make the ORB features scale-invariant, the ORB-SLAM3 algorithm downsamples the original image to obtain the image pyramid, as shown in Figure 10, and extracts the ORB feature points from the images on each pyramid layer. As the size of the images on each pyramid is different, feature points must be assigned to the images on different layers. The number of assigned feature points is calculated according to the scaling ratio and total feature points, as shown in Equation (7), where W and H are the width and height of the image, respectively; the scaling factor of the image pyramid β is 1.2; S(n) is the area of each layer of the pyramid image; the number of pyramid layers n is 8; St(n) is the total area of all the pyramid Images; Nua is the number of feature points to be extracted per unit area of the image, and the total number of feature points N is 1000; and N(i) is the number of feature points allocated to the ith layer of the pyramid image.

(7) $$S(i)=H\times W\times \beta^{\left(i-1\right)}\;St(n)=H\times W\times \beta^{0}+H\times W\times \beta^{1}+\;\cdots +H\times W\times \beta^{\left(n-1\right)}\;=\frac{W\times H\times (1-\beta^{n})}{1-\beta }\;Nua=\frac{N}{St(n)}=\frac{N\times (1-\beta )}{W\times H\times (1-\beta^{n})}\;N(i)=Nua\times S(i)=\frac{N\times (1-\beta )}{1-\beta^{n}}\times \beta^{\left(i-1\right)}$$

**Figure 11:**
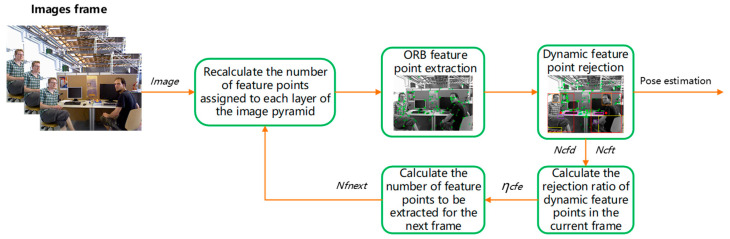
Dynamic feature point compensation strategy process.

Considering that the dynamic environment poses a considerable challenge to the localization accuracy of the VSLAM algorithm, in highly dynamic scenes, many dynamic feature points are rejected, which leads to an insufficient number of feature points used for the camera pose estimation, resulting in a decrease in accuracy or a loss of pose tracking. To compensate for the rejected dynamic feature points, and in accordance with the principle of not adding too many feature points as much as possible, the number of feature points to be extracted in the next frame is updated in real time by calculating the ratio of the number of rejected dynamic feature points to the initial total number of feature points in each frame, as shown in Figure 11.

(8) $$\eta_{cfe}=\frac{N_{cfd}}{N_{cft}}\times 100\%\;N_{fnext}=\frac{N}{\left(1-\eta_{cfe}\right)}$$

(9) $$N_{fnext}=\frac{N}{\left(1-\frac{N_{cfd}}{N_{cft}}\right)}$$

In Equations (8) and (9), $\eta_{cfe}$ represents the elimination ratio of the dynamic feature points in the current image frame, and this equation represents the ratio of the eliminated dynamic feature points in the current frame to the total feature points. $N_{cfd}$ represents the number of dynamic feature points removed from the current frame, and $N_{cft}$ represents the total feature points extracted from the current frame. N represents the initial total number of feature points and is set to 1000. $N_{fnext}$ represents the total number of feature points to be extracted in the next frame, and $1-\eta_{cfe}$ represents the retention ratio of static feature points.

Considering that numerous dynamic feature points are eliminated in highly dynamic scenarios, when the elimination ratio of dynamic feature points is too large, the VSLAM algorithm is prone to pose loss due to an insufficient number of feature points. Therefore, when the elimination ratio of dynamic feature points is greater than 50%, a coefficient greater than 1 is added to the above equation to obtain more static feature points (see Equation (10)). The value of coefficient α is greater than 1, and ε is the self-defined upper limit of the total feature points. The values of α and ε can be set according to the actual situation and the performance of the operating equipment. In this study, α was set to 1.2, and ε was set to 3500.

(10) $$N_{fnext}=\frac{N}{\left(1-\frac{N_{cfd}}{N_{cft}}\right)}\times \alpha \;N_{fnext}<\varepsilon$$

The total number of fixed feature points N extracted by ORB-SLAM3 can be replaced with the calculated $N_{fnext}$ (see Equation (11)). Through the above methods, the number of feature points extracted by the algorithm in this paper can be adjusted adaptively according to the complexity of dynamic objects in the scene.

(11) $$Nua=\frac{N_{fnext}}{St(n)}=\frac{N_{fnext}\times (1-\beta )}{W\times H\times (1-\beta^{n})}\;N\left(i\right)=Nua\times S\left(i\right)=\frac{N_{fnext}\times (1-\beta )}{1-\beta^{n}}\times \beta^{\left(i-1\right)}$$

#### 3.5.3. ‘Hand’ Peripheral Feature Point Culling

The size of a small depth object in a potentially dynamic object is small, and it will be difficult to detect or blocked by the hand, resulting in the problem that some dynamic feature points are not eliminated. To solve the above problem, the ‘hand’ box can be expanded to twice the original circumference. The feature points in the range are excluded and judged. The depth value of the hand is obtained firstly, and then the depth range of the feature points to be eliminated is calculated according to the maximum depth of the small depth object and the depth value of the hand, which is shown in Equation (12). Dextmin and Dextmax represent the minimum and maximum depth values of feature points to be rejected in the extended range of ‘hand’, respectively. When the depth of the feature point in the extended box is within the depth range, the feature point is regarded as a dynamic feature point and rejected.

(12) $$Dextmin={Dhand}^{\prime }+\frac{Dhand}{2}-Dsmall\;Dextmax={Dhand}^{\prime }+\frac{Dhand}{2}+Dsmall\;[Dextmin,Dextmax]$$

#### 3.5.4. Full Rejection of Complex Dynamic Feature Points

Dynamic environments are very challenging for the VSLAM algorithm. To improve the robustness of this algorithm, the proposed approach adopts the full rejection strategy for the feature points in the dynamic detection box in some special cases, as follows:

(1) Because the method proposed in this paper combines YOLOv5 and depth information to refine the exclusion range of the feature points in the dynamic detection box, the failure of image depth information will affect the pose estimation of YDD-SLAM. To improve the robustness of YDD-SLAM, when the depth information fails, the feature points in the dynamic object detection box are eliminated completely. For example, if the object detected exceeds the depth detection range of the depth camera, the depth information of part of the image will fail. When the depth of this part of the image must be used to determine whether the feature points in the object box are deleted, the feature points in the object box will be completely eliminated.
(2) When the ratio of the detection box areas of ‘person’ and ‘automatic objects’ to the total area of the image is too small, such as if the ‘person’ is in the distance or partially obscured, the detection box will be too small. At this time, the number of static feature points in the box is small, and the accuracy of the depth value of the distant person is not high. To improve the real-time performance and robustness of YDD-SLAM, a rejection judgment is skipped for the feature points in the detection boxes of ‘person’ and ‘automatic objects’ with excessively small areas, and the full rejection strategy is implemented.
(3) When the coincidence degree of the ‘person’ box is greater than 50%, the information in the ‘person’ box is too redundant and leads to information matching difficulties. Therefore, a total elimination strategy is adopted for the feature points in the ‘person’ box in this case.

## 4. Experimental Results and Discussion

This section will demonstrate the effectiveness of the YDD-SLAM algorithm in dynamic scenarios, present relevant experiments conducted on the public TUM dataset [43], and compare the proposed algorithm with ORB-SLAM3 [11] and various types of dynamic VSLAM algorithms. Finally, real dynamic scenario tests are presented to verify the effectiveness and robustness of the YDD-SLAM algorithm. The data obtained using the proposed algorithm that are presented in the experimental tables are the mean values calculated by five repetitive experiments, and the equipment used for the text experiments was a Lenovo R7000P laptop equipped with an AMD Ryzen 7 4800H CPU model, 16 GB of running memory (RAM), and an NVIDIA RTX 2060 graphics card. The running environment of the algorithm is Ubuntu20.04, the ROS system version used is Noetic, and the depth camera used in the actual scene test is ORBBEC Astra.

### 4.1. Performance of YDD-SLAM

**Figure 12:**
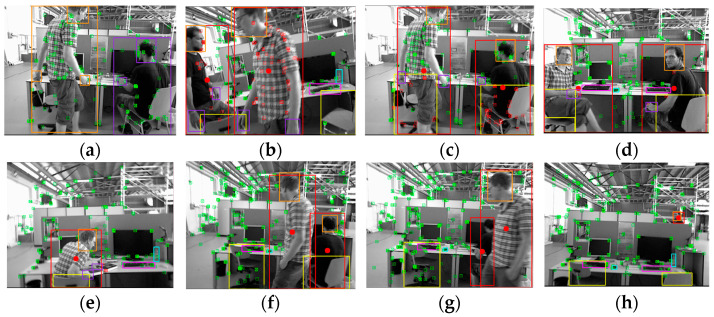
YDD-SLAM dynamic scene effects. Rectangles with different colors are object detection results for different objects. The circle is the ‘person’ box center of the YDD SLAM algorithm. The small green squares represent the feature points. (**a**) Matching of ‘person’, ‘head’, and ‘hand’ information. (**b**) ‘Human body’ dynamic feature point culling. (**c**) ‘Potentially dynamic objects’ dynamic feature point culling. (**d**) Edge feature point rejection. (**e**) Feature points around the hand are eliminated. (**f**) Less than 50% coincidence of ‘person’ box. (**g**) ‘Person’ box coincidence greater than 50%. (**h**) The feature points of the small area ‘person’ box are all eliminated.

The YDD-SLAM algorithm integrates ORB-SLAM3 with object detection and depth information and divides indoor objects into eight subcategories. The specific effect is depicted in Figure 12. Firstly, the information for ‘person’, ‘head’, and ‘hand’ is matched, as shown in Figure 12a. Secondly, the feature points of dynamic objects and moving potentially dynamic objects are eliminated. As can be seen from Figure 12b,c, the recognition effect of the proposed algorithm on dynamic feature points is close to the segmentation effect. Finally, this paper proposes a series of feature point optimization strategies for indoor dynamic scenes, as depicted in Figure 12d–h. As can be seen from Figure 12d, the edge feature point elimination strategy proposed in this paper can effectively eliminate unstable feature points on the edges of dynamic objects. As demonstrated by Figure 12e, the strategy used to eliminate dynamic feature points around the hand can effectively eliminate some unrecognized dynamic feature points.

**Figure 13:**
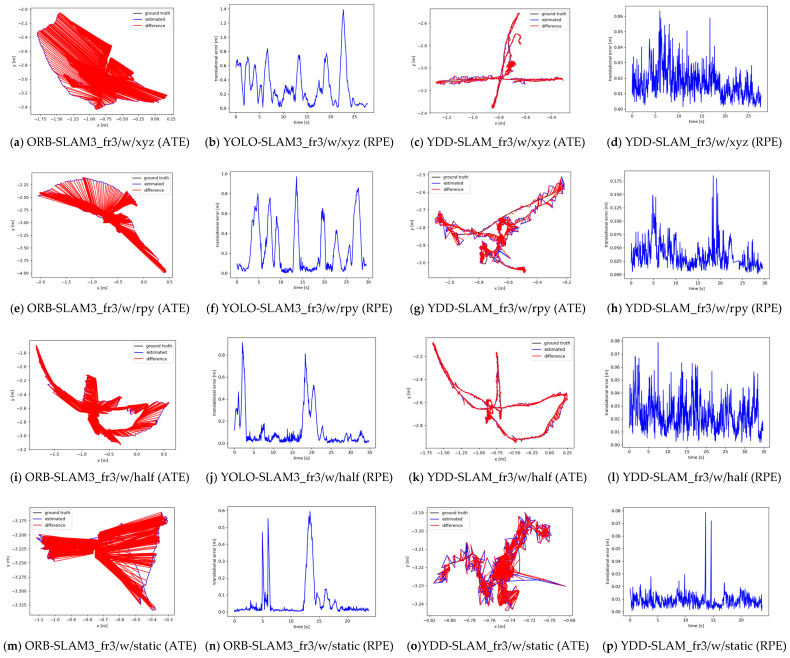

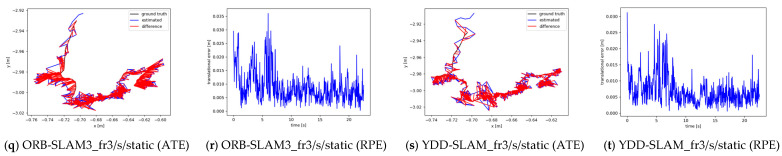
Comparison of absolute trajectory error and relative pose error between ORB-SLAM3 and YDD-SLAM. The horizontal and vertical coordinates of ATE are in units of meters. Black indicates the ground truth, blue indicates the trajectory estimated in this paper, and red indicates the error. The horizontal axis of RPE is time (s), and the vertical axis indicates the translation error (m).

The YDD-SLAM algorithm proposed in this paper represents an improvement compared to the ORB-SLAM3 algorithm, as it is validated by selecting a dynamic scene TUM dataset with four fr3_walking sequences and one fr3_sitting sequence. The dataset was obtained in an indoor office scene, containing common indoor objects such as people, chairs, keyboards, cups, and books. The people in the fr3_walking sequence dataset were in the walking state most of the time and in the sitting state for a small portion of time. The person in the fr3_sitting sequence dataset selected in this paper was always in the sitting state, and only the hand and head were moving. The absolute trajectory error (AET) and relative pose error (RPE) are two error metrics commonly used to evaluate the accuracy of VSLAM algorithms. The ATE can measure the global consistency of the trajectory well, and the RPE is well-suited to measure the drift of pose translation and rotation. In this paper, the root-mean-square error (RMSE), mean (Mean), median (Median), and standard deviation (S.D.) of the ATE and RPE are used to demonstrate the accuracy of the VSLAM algorithm. As shown in Figure 13, a longer red line in the ATE plot indicates a larger ATE of the algorithm, and a larger value in the RPE plot represents a larger RPE.

As can be seen from Figure 13 and Table 3, Table 4 and Table 5 compared with ORB-SLAM3, the YDD-SLAM algorithm achieves greatly improved accuracy in the dynamic scenario, especially in the fr3_walking sequence TUM data set. The improvement effect is the most obvious in this case, and the improvement effect of the ATE is basically above 90%, with the maximum improvement effect reaching 98%. However, according to the results for the fr3_sitting sequence data set, compared with ORB-SLAM3, the algorithm proposed in this paper exhibits no obvious improvement, because the human body was treated as a dynamic object in this study. Thus, any feature point of the human body was considered as a dynamic feature point and was removed. Consequently, the static feature points of the human body were also eliminated, resulting in a lower pose estimation accuracy of the proposed algorithm in the low-dynamic scene with the human body.

### 4.2. Comparison of YDD-SLAM-Derived Algorithms

As described at the beginning of this paper, the YOLOv5 algorithm was used to eliminate all the dynamic feature points in the ‘person’ box and was named YOLO-SLAM3. Then, combined with the depth information, the range of dynamic feature points in the ‘person’ box was refined to obtain Person-SLAM. Finally, the YDD-SLAM algorithm was proposed. The YOLO-SLAM3 and Person-SLAM algorithms are the intermediate steps towards the YDD-SLAM algorithm.

**Figure 14:**
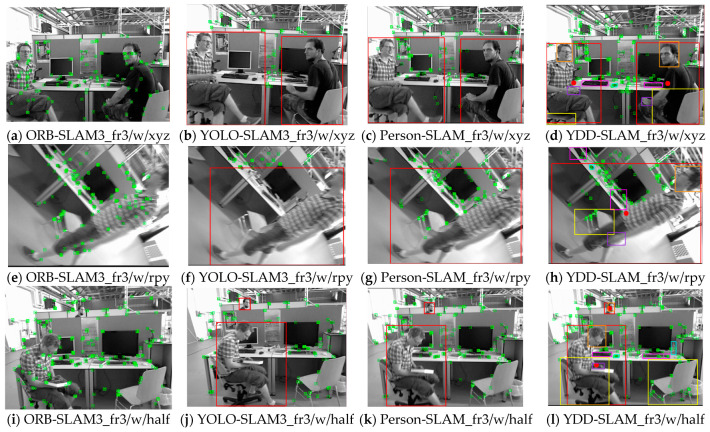
Effect of different algorithms in a dynamic environment. Rectangles with different colors are object detection results for different objects. The circle is the ‘person’ box center of the YDD SLAM algorithm. The small green squares represent the feature points.

In this part of the experiment, the above three algorithms were compared with ORB-SLAM3, and the operation effects of three fr3_walking sequence TUM data sets were selected for demonstration, as shown in Figure 14. As can be seen from the figure, ORB-SLAM3 does not remove the dynamic feature points of the human body in a dynamic environment. YOLO-SLAM3 removes all feature points in the ‘person’ box, but also removes static feature points in the box. Person-SLAM combines depth information on the basis of YOLO-SLAM3, retaining static feature points in the ‘person’ box as much as possible, but does not consider other classes of objects. YDD-SLAM considers potentially dynamic objects on the basis of Person-SLAM and adds several optimization strategies for the feature points of a dynamic environment.

**Figure 15:**
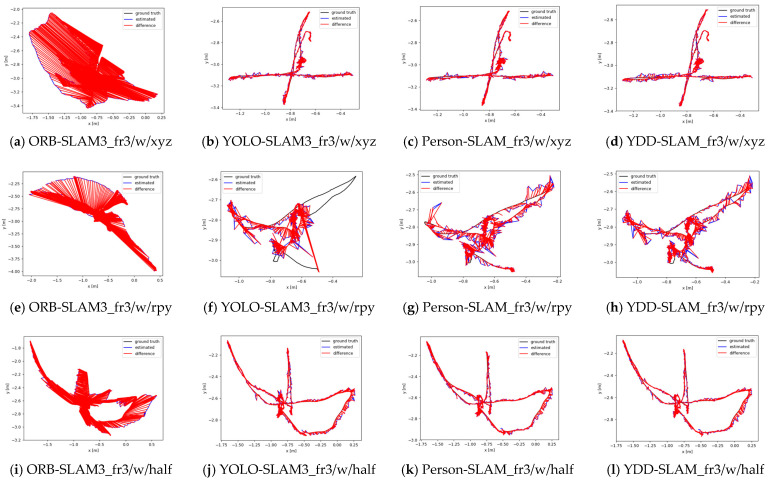
Comparison of ATEs of different algorithms. The horizontal and vertical coordinates of ATE are in units of meters. Black indicates the ground truth, blue indicates the estimated trajectory, and red indicates the error.

Figure 15 compares the absolute trajectory errors of the above four VSLAM algorithms in TUM datasets of three fr3_walking sequences. This figure demonstrates that the absolute trajectory errors of ORB-SLAM3 are very large, whereas the absolute trajectory errors of the other three dynamic VSLAM algorithms are much lower than that of ORB-SLAM3. As can be seen from Table 6, Table 7 and Table 8 the pose estimation results of the YDD-SLAM algorithm in the three dynamic TUM datasets are all the best, followed by the results of Person-SLAM and those of YOLO-SLAM3 and ORB-SLAM3, which also verifies the effectiveness and robustness of the proposed YDD-SLAM algorithm.

### 4.3. Comparison of Mainstream Dynamic VSLAM

**Figure 16:**
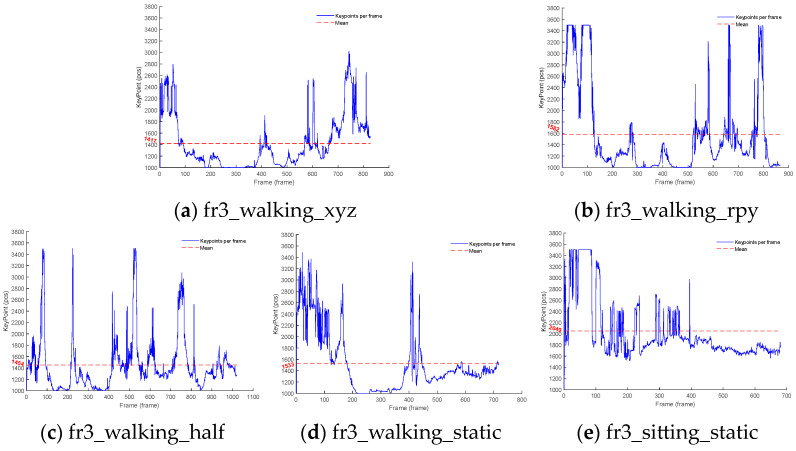
Number of feature points to be extracted per image frame for each dataset. The horizontal axis represents Frame (frame), and the vertical axis shows KeyPoint (pcs). The red dotted line indicates the mean value of KeyPoints, and blue indicates KeyPoint per frame.

When too many dynamic feature points are eliminated in a highly dynamic scene, the pose estimation accuracy of the VSLAM algorithm is degraded, which causes tracking failure; to address this problem, YDD-SLAM adds a feature point compensation strategy to attempt to ensure that the number of static feature points left after the dynamic feature points of each image frame are eliminated is still approximately 1000. As can be seen in Figure 16, the number of feature points extracted from a small number of image frames is limited to a maximum of 3500. When the number of feature points to be extracted from the next image frame is 1000, the current scene is considered to be a static scene. In Figure 16a–d, the human body in the dataset exists in the field of view of the camera most of the time and disappears only for a small portion of time. In Figure 16e, the number of feature points to be extracted in the next frame is above 1000, which indicates that the human body always exists in the dataset; therefore, the average number of feature points to be extracted is high.

In this study, several excellent Dynamic VSLAM algorithms developed in recent years were selected and compared with YDD-SLAM. Dynamic-VINS [33] combines object detection and depth information to eliminate dynamic feature points in an environment with limited computing resources. RS-SLAM [39], DS-SLAM [44], and Det-SLAM [31] all adopt segmentation methods to eliminate dynamic feature points. The RMSE of the ATE of each algorithm was compared to evaluate the advantages and disadvantages of the proposed algorithm. As can be seen from Table 9, the RMSE of YDD-SLAM is superior to those of the other algorithms for the fr3_walking_rpy and fr3_walking_half sequences, because the dynamic feature point compensation strategy of YDD-SLAM can improve its robustness in highly dynamic scenarios. The RMSEs for the other sequences are close to the optimal results of most segmentation algorithms, which verifies the effectiveness and robustness of YDD-SLAM.

Table 10 shows the median and average tracking time of each frame of the TUM dataset run by YDD-SLAM under CPU and combined CPU and GPU environments, respectively. The table demonstrates that in the CPU environment, the median and average image tracking time per frame using YDD-SLAM are between 68.9 and 70.7 ms, whereas in the CPU + GPU environment, the median and average image tracking time per frame are only between 14.7 and 17.3 ms, meeting the real-time requirements.

The time data for the YDD-SLAM and ORB-SLAM3 algorithms refers tp the average time of running five TUM datasets. All the SLAM algorithms compared in this paper can meet the real-time requirements [33,39,44]. As can be seen from Table 11, the algorithms in this paper have the shortest time consumption for both the dynamic feature point processing time and the image processing time for deep learning, while the image tracking time per frame is only slightly lower than the ORB-SLAM3 algorithm. Since the YOLOv5 target detection algorithm proposed in this paper is an independent module and takes less time than the per-frame image tracking time of the SLAM algorithm, the target detection algorithm basically does not affect the running time of the main thread of the SLAM algorithm. Combining the above analysis of accuracy and time, the YDD-SLAM algorithm proposed in this paper has excellent accuracy and real-time meets the use of requirements.

The memory overhead and CPU and GPU consumption of the YDD-SLAM algorithm and its related algorithms are given in Table 12, where the tabular data is the average value of running five TUM datasets. From the table, it can be seen that the memory overhead of the YOLOv5 algorithm is larger than that of the ORB-SLAM3 algorithm, and when only the CPU is used to run the YOLOv5 or YDD-SLAM algorithms, the CPU resource consumption is larger. In the CPU and GPU mutual cooperation mode, running YOLOv5 or YDD-SLAM algorithm, the CPU occupancy is only 16.6%, while the GPU occupies about 1 GB. In this mode, the algorithm proposed in this paper can achieve high real-time performance while occupying less CPU and GPU resources.

### 4.4. Actual Scenario Verification

**Figure 17:**
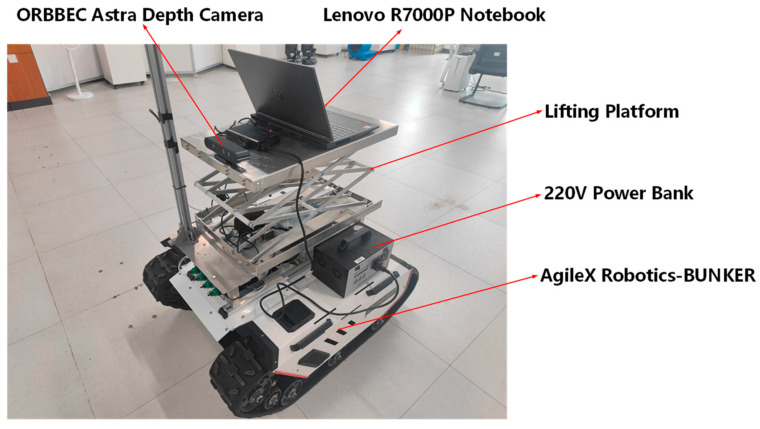
Experimental equipment platform.

To verify the practical application effect of YDD-SLAM, experiments were conducted on the mobile robot shown in Figure 17. The mobile platform used was Agilex Robotics-BUNKER, which was equipped with a 220 V mobile power supply, lift platform, Lenovo R7000P laptop, and ORBBEC Astra depth camera.

**Figure 18:**
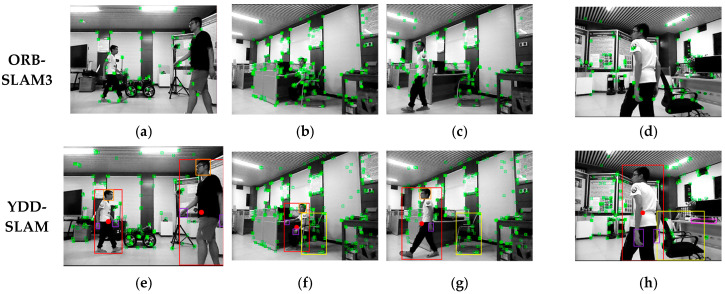
Running effect in actual dynamic scenarios. (**a**–**d**) Effects of ORB-SLAM3 algorithm. (**e**–**h**) Effects of YDD-SLAM algorithm. Rectangles with different colors are object detection results for different objects. The circle is the ‘person’ box center of the YDD SLAM algorithm. The small green squares represent the feature points.

**Figure 19:**
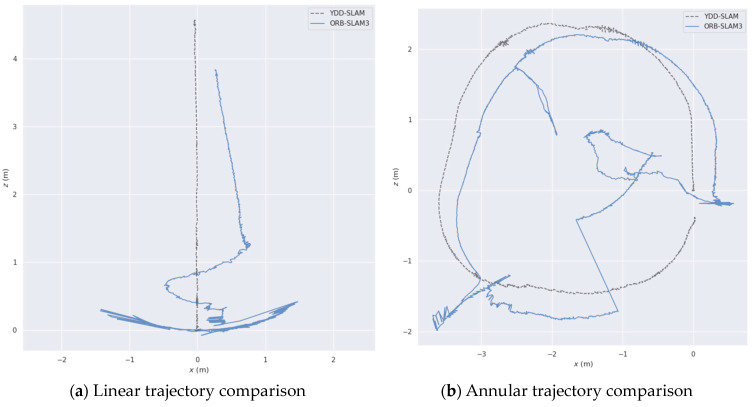
Comparison of driving trajectories of different shapes for real dynamic scenes.

The experimental environment was a combination of office and laboratory settings, accompanied by the movement of personnel, as shown in Figure 18. ORB-SLAM3 and YDD-SLAM were compared in the same scene and under identical conditions, and the mobile robot was controlled by remote control in the linear and annular directions separately. Figure 18a–d depict the operation effect of ORB-SLAM3. Evidently, the feature points on dynamic objects are not removed. Figure 18e–h demonstrate the operation effect of YDD-SLAM. The proposed algorithm exhibits an excellent ability to eliminate dynamic feature points. Further, while eliminating dynamic feature points in the detection box, static feature points are retained as much as possible. Figure 19 depicts the pose estimation trajectories of the two algorithms for the linear and annular driving trajectories in the above actual dynamic environment. As can be seen from Figure 19a,b, ORB-SLAM3 produces large errors in the pose estimation of straight line or annular driving trajectories in the actual dynamic environment. Meanwhile, YDD-SLAM can perform the pose estimation well in the dynamic environment, and the trajectory shape estimated by the proposed algorithm is basically consistent with the driving trajectory shape of the mobile robot. Some local tracks in the figure have slight fluctuations. Due to the unstable structure of the lifting platform used in this study, the camera shook when the traveling speed changed rapidly. In the actual dynamic environment, the effectiveness and robustness of YDD-SLAM were further verified by comparing the operation effect of ORB-SLAM3 and the trajectory estimation results of the pose. From Table 13, it can be seen that the median and mean tracking times of YDD-SLAM for each frame of pictures are between 0.0557 and 0.0566 s in the CPU environment. In the CPU+GPU environment, the tracking times of the algorithm proposed in this paper are very close to those of the ORB-SLAM3 algorithm for each frame picture, which verifies that the proposed algorithm meets the real-time performance requirements.

## 5. Conclusions

Most dynamic VSLAM algorithms cannot achieve sufficient positioning accuracy and real-time performance simultaneously; further, the time cost of using segmentation to remove dynamic feature points is too high. To solve these problems, this paper proposes an improved YDD-SLAM algorithm based on ORB-SLAM3. The algorithm proposed in this paper aims to solve the VSLAM problem in indoor dynamic scenes, which is applicable to a variety of typical indoor scenes, and this paper mainly focuses on the office scene as the introductory object. This algorithm introduces the YOLOv5 object detection algorithm, which takes a short time; the algorithm combines the depth information to achieve the fine elimination of dynamic feature points in the detection box, and achieves an elimination effect close to image segmentation. Firstly, the algorithm divides common indoor objects into eight small categories according to their motion characteristics and depth values and uses the YOLOv5 object detection algorithm to identify these categories. Secondly, the three subclasses of ‘person’, ‘head’, and ‘hand’ are matched according to the geometry and depth information, and the depth range of ‘human body’ is finally calculated to judge the dynamic feature points. The depth information in the dynamic detection box is fully utilized and the static feature points are retained as much as possible to improve the accuracy and robustness of the pose estimation of the VSLAM algorithm. Simultaneously, the depth and geometry information of the dynamic object are used to judge a potentially dynamic object that may move, and its feature points are removed. Then, optimization methods for dynamic feature points are proposed, such as an edge feature point elimination strategy and a dynamic feature point compensation strategy. Finally, compared to the pose estimation accuracy of the ORB-SLAM3 algorithm, the pose estimation accuracy of YDD-SLAM is increased by up to 98%. Based on the comparison of the proposed algorithm with other excellent dynamic VSLAM algorithms, the accuracy of the proposed algorithm is close to the optimal results of most algorithms, and the RMSE results for the fr3_walking_rpy and fr3_walking_half sequences are better than those of the other algorithms. These findings further verify the effectiveness and robustness of the proposed algorithm.

Because the human body has always been regarded as a dynamic object, in a dynamic scene, when the human body is in a stationary sitting posture, the proposed algorithm still removes its feature points. The experiments also demonstrated that in some scenes, the algorithm sometimes misses the object class in a certain frame image. In dynamic scenes, dynamic targets are the regions of interest and the objects we focus on processing. Visual attention may help us to better detect the region of interest and improve the processing effect on dynamic target objects; further, introducing audio information into visual attention can effectively improve the performance of gaze prediction [45,46,47]. Information security is vital for a system to prevent system failure due to malicious intrusion [48]. Therefore, the topics for future work are as follows:

(1) The feature points in the dynamic depth range will be further evaluated by adding methods such as Epipolar Geometry or optical flow.
(2) To avoid missing detection in the object detection algorithm, the missing detection will be processed by the speed information of the image frame or the IMU information.
(3) Dynamic VSLAM algorithms for audio information and visual attention will be introduced to further deal with more interesting dynamic target regions.
(4) Various means will be combined to cryptographically protect the data information of the dynamic VSLAM algorithm.

## Figures and Tables

**Table 1:** Advantages and disadvantages of common methods for dynamic SLAM algorithms.

Sequences	Geometric Method	Deep Learning Method	
		Image Segmentation	Object Detection
Advantages	Dynamic feature points in the environment can be eliminated well.	Accurate dynamic feature point rejection	Good real-time performance
Disadvantages	The removal of dynamic feature points is general and too dependent on thresholds.	Poor real-time performance	Static feature points are over-rejected.

**Table 2:** Constant values (m).

Dhead	Dhand	Dhalfhand	Dsmall	Dmedium	Dlarge
0.2	0.18	0.5	0.2	0.45	0.7

**Table 3:** Results of comparison between ORB-SLAM3 and YDD-SLAM algorithm (ATE) (Unit: m).

Sequences	ORB-SLAM3				YDD-SLAM				Improvements (%)			
	RMSE	Mean	Median	S.D.	RMSE	Mean	Median	S.D.	RMSE	Mean	Median	S.D.
fr3_w_xyz	0.6930	0.5810	0.4500	0.3742	0.0151	0.0132	0.0117	0.0075	97.82	97.73	97.40	98.00
fr3_w_rpy	0.6381	0.5468	0.5151	0.3263	0.0355	0.0287	0.0230	0.0208	94.44	94.75	95.54	93.63
fr3_w_half	0.3196	0.2910	0.2667	0.1272	0.0275	0.0241	0.0217	0.0132	91.40	91.72	91.86	89.62
fr3_w_static	0.3274	0.3010	0.2999	0.1207	0.0082	0.0067	0.0060	0.0047	97.50	97.77	98.00	96.11
fr3_s_static	0.0108	0.0093	0.0082	0.0054	0.0067	0.0059	0.0052	0.0033	37.96	36.56	36.59	38.89

**Table 4:** Results of comparison between ORB-SLAM3 and YDD-SLAM algorithm (RPE translation part) (Unit: m).

Sequences	ORB-SLAM3				YDD-SLAM				Improvements (%)			
	RMSE	Mean	Median	S.D.	RMSE	Mean	Median	S.D.	RMSE	Mean	Median	S.D.
fr3_w_xyz	0.0251	0.0201	0.0161	0.0150	0.0119	0.0097	0.0081	0.0068	52.59	51.74	49.69	54.67
fr3_w_rpy	0.0286	0.0217	0.0167	0.0185	0.0212	0.0153	0.0120	0.0147	25.87	29.49	28.14	20.54
fr3_w_half	0.0227	0.0162	0.0120	0.0158	0.0142	0.0116	0.0098	0.0082	37.45	28.40	18.33	48.10
fr3_w_static	0.0187	0.0103	0.0067	0.0154	0.0072	0.0055	0.0046	0.0046	61.50	46.60	31.34	70.13
fr3_s_static	0.0058	0.0050	0.0043	0.0031	0.0054	0.0043	0.0037	0.0030	6.90	14.00	13.95	3.23

**Table 5:** Results of comparison between ORB-SLAM3 and YDD-SLAM algorithm (RPE rotation part) (unit-less).

Sequences	ORB-SLAM3				YDD-SLAM				Improvements (%)			
	RMSE	Mean	Median	S.D.	RMSE	Mean	Median	S.D.	RMSE	Mean	Median	S.D.
fr3_w_xyz	0.0148	0.0117	0.0096	0.0091	0.0095	0.0067	0.0056	0.0067	35.81	42.74	41.67	26.37
fr3_w_rpy	0.0162	0.0131	0.0110	0.0095	0.0122	0.0090	0.0072	0.0083	24.69	31.30	34.55	12.63
fr3_w_half	0.0187	0.0109	0.0089	0.0140	0.0102	0.0087	0.0075	0.0054	45.46	20.18	15.73	61.43
fr3_w_static	0.0091	0.0060	0.0045	0.0067	0.0046	0.0038	0.0033	0.0026	49.45	36.67	26.67	61.19
fr3_s_static	0.0114	0.0036	0.0030	0.0022	0.0039	0.0032	0.0027	0.0021	7.14	11.11	10.00	4.55

**Table 6:** RMSE comparison results of different algorithms used in this study (ATE) (Unit: m).

Sequences	ORB-SLAM3	YOLO-SLAM3	Person-SLAM3	YDD-SLAM
fr3_walking_xyz	0.6930	0.0166	0.0162	**0.0151**
fr3_walking_rpy	0.6381	0.0607	0.0414	**0.0355**
fr3_walking_half	0.3196	0.0341	0.0308	**0.0275**

**Table 7:** RMSE comparison results of different algorithms used in this study (RPE translation part) (Unit: m).

Sequences	ORB-SLAM3	YOLO-SLAM3	Person-SLAM3	YDD-SLAM
fr3_walking_xyz	0.0251	0.0130	0.0124	**0.0119**
fr3_walking_rpy	0.0286	0.0301	0.0265	**0.0213**
fr3_walking_half	0.0227	0.0175	0.0153	**0.0142**

**Table 8:** RMSE comparison results of different algorithms used in this study (RPE rotation part) (unit-less).

Sequences	ORB-SLAM3	YOLO-SLAM3	Person-SLAM3	YDD-SLAM
fr3_walking_xyz	0.0148	0.0099	0.0097	**0.0095**
fr3_walking_rpy	0.0162	0.0235	0.0139	**0.0122**
fr3_walking_half	0.0187	0.0107	0.0105	**0.0102**

**Table 9:** Comparison of the RMSEs of different algorithms (ATE) (Unit: m).

Sequences	ORB-SLAM3	Dynamic-VINS [33]	RS-SLAM [39]	DS-SLAM [44]	Det-SLAM [31]	YDD-SLAM (Ours)
fr3_walking_xyz	0.6930	0.0486	**0.0146**	0.0247	0.0482	0.0151
fr3_walking_rpy	0.6381	0.0629	0.1869	0.4442	0.0389	**0.0355**
fr3_walking_half	0.3196	0.0608	0.0425	0.0303	0.0925	**0.0275**
fr3_walking_static	0.3274	0.0077	0.0067	0.0081	**0.0017**	0.0082
fr3_sitting_static	0.0108	-	0.0066	0.0065	**0.0036**	0.0067

**Table 10:** Tracking time (ms) per frame of the TUM dataset when running the YDD-SLAM algorithm.

Sequences	fr3_walking_xyz	fr3_walking_rpy	fr3_walking_half	fr3_walking_static	fr3_sitting_static
Times-Median (CPU)	69.5404	69.5308	70.0303	69.9896	69.9784
Times-Mean (CPU)	69.8520	70.0031	70.6693	70.4850	69.1083
Times-Median (CPU + GPU)	15.7447	16.0031	16.7050	14.7780	15.3516
Times-Mean (CPU + GPU)	16.3400	16.2513	17.2688	15.8070	15.9183

**Table 11:** Time comparison (ms) of YDD-SLAM algorithm with various types of algorithms.

SLAM Algorithm	Dynamic Feature Point Processing	Deep Learning	Tracking Time per Frame	Experimental Platforms
RS-SLAM [39]	-	158.13	-	Inter i5-7500 CPU Nvidia GTX 1060 GPU (RAM 16 GB)
DS-SLAM [44]	29.5087	37.5333	59.4	Inter i7 CPU P 4000 GPU (RAM 32 GB)
Dynamic-VINS [33]	1.3424	17.5850	19.8980	HUAWEI Atlas200 DK
ORB-SLAM3	-	-	15.6624	AMD R7 4800H CPU Nvidia GTX 2060 GPU (RAM 16 GB)
YDD-SLAM	0.9132	11.5	16.3171	AMD R7 4800H CPU Nvidia GTX 2060 GPU (RAM 16 GB)

**Table 12:** Memory footprint and resource usage of various types of algorithms. VIRT denotes virtual memory, RES denotes resident memory, and SHR denotes shared memory.

Algorithms	VIRT (MB)	RES (MB)	SHR (MB)	CPU (%)	GPU (MB)
ORB-SLAM3 (CPU)	2740.60	673.40	101.40	11.88	-
YOLOv5 (CPU)	9789.44	1550.00	405.20	49.80	-
YDD-SLAM (CPU)	12,546.44	2192.20	507.80	61.06	-
YOLOv5 (CPU+GPU)	12,185.60	2827.40	684.40	3.56	1054.80
YDD-SLAM (CPU+GPU)	14,886.40	3496.80	786.80	16.60	1054.80

**Table 13:** Tracking times (ms) per frame of the YDD-SLAM and ORB-SLAM3 algorithms.

Sequences	ORB-SLAM3/Linear	YDD-SLAM/Linear	ORB-SLAM3/Annular	YDD-SLAM/Annular
Times-Median (CPU)	14.5005	56.6352	15.9373	56.1799
Times-Mean (CPU)	15.4729	56.3241	17.0308	55.7422
Times-Median (CPU + GPU)	-	14.5367	-	14.3323
Times-Mean (CPU + GPU)	-	15.1504	-	14.8671

## Data Availability

The dataset used in this paper is the public TUM dataset. The download address is as follows: https://vision.in.tum.de/data/datasets/rgbd-dataset (accessed on 23 June 2023).
